# Design and construction of an integrated tetrafluoroethane (R134a) refrigerator-waste heat recovery dryer for fabric drying in tropical regions

**DOI:** 10.1016/j.heliyon.2020.e04838

**Published:** 2020-09-02

**Authors:** Ebenezer I. Onyeocha, Kevin N. Nwaigwe, Nnamdi V. Ogueke, Emmanuel E. Anyanwu

**Affiliations:** a Department of Mechanical Engineering, Federal University of Technology Owerri, PMB 1526 Owerri Nigeria; b Department of Mechanical Engineering, University of Botswana, Gaborone, Botswana

**Keywords:** Energy, Mechanical engineering, Energy conservation, Energy economics, Heat transfer, Mass transfer, Mechanical systems, Waste heat recovery, Refrigeration, Drying chamber, Vapour compression cycle, Heat pump, Temperature

## Abstract

A work on the design and construction of an integrated tetrafluoroethane (R134a) refrigerator-waste heat recovery dryer suitable for use in tropical regions is presented. The system comprises of a refrigerator with its condenser unit retrofitted to serve as the heat recovery mechanism and a drying chamber. The refrigerator had a vapour compression cycle driven by environmentally friendly R134a working fluid (refrigerant). The dryer component was powered by heat dissipated by the condenser piping from the exit of the compressor (superheat region) to the entrance of the sub-cooled region. The maximum drying temperature attained during pre-loading test was 49 °C while the evaporator provided cooling at a temperature of 5 °C. The specific moisture extraction rate of the dryer varied over 0.19–0.22 kg/kW.hr while 150W of cooling was produced at the evaporator in all cases. The energy utilization ratio obtained was 0.92, indicating that 92% of the waste heat recovered was actually utilized. The system coefficient of performance was estimated to be 10.09 thus indicating that the energy derived from IRWHRD was 10 times the energy it consumed. Application potentials therefore exist for use of this dual purpose system for simultaneous production of refrigeration and heating. Storage of food and drying of fabrics make the IRWHRD an option for use in both agricultural development and entrepreneurship development in laundry business.

## 1 Introduction

Re-use of waste energy such as rejected heat from a thermodynamic system is an energy conservation measure. Heat recovery technology provides a means to effectively recover waste heat and convert it to useful purposes, thus offsetting the fuel that could have been utilized. Hence, heat recovery leads to energy savings and operational cost reduction. Indirect benefits of heat recovery include reduction in equipment sizes, reduction in pollution, and reduction in auxiliary energy consumption [1]. Many industrial, commercial and institutional use of energy results in excessive release of waste heat to the environment. Approximately up to 20% of input energy is lost in the flue gas [2]. There have been several studies on harnessing waste heat and converting it into useful energy. The overall aim of such studies is to improve the efficiency of the waste heat recovery system either by careful selection of working fluid, parameter balancing or process improvement practices. Waste heat energy can be recovered from a system using various methods. They include the organic Rankine cycle (ORC), the Brayton cycle, thermoelectric generator, Kalina Rankine cycle, steam Rankine cycle, and many other advanced technologies [3, 4, 5, 6, 7, 8, 9, 10, 11, 12, 13]. Organic Rankine cycle is renowned for its use of organic fluids as working medium instead of steam. It is capable of converting heat at lower temperatures below boiling point of water and its use is increasingly popular for waste heat recovery systems. A steady-state thermodynamic model for an ORC system usually consists of an evaporator, an expansion device, a condenser, a pump and a superheater [4]. Ahmed et al [14] studied design methodology of organic Rankine cycle for waste heat recovery in cement plants. R134a was chosen for the design due to its low ozone depletion potential (ODP) value of zero and global warming potential (GWP) value of 1300, as against the very high values for most other organic fluids. The study concluded that that the best operating conditions for R134a existed when the turbine inlet temperature was as low as between 120 °C and 220 °C. Under this condition, the heat exchanger effectiveness was increased up to 93%.

Several other studies have been undertaken on the utilization of various vapour compression and refrigeration systems for waste heat recovery. Panesar [15] investigated an innovative system for integrated cooling and heat recovery in automotive heavy duty diesel engines. The work proposed the use of a cascade hence allowing exhaust heat recovery and complete coolant heat recovery even though it applied organic Rankine cycle approach. Oluleye et al [16] evaluated the potentials of waste heat recovery from process sites. Using a refinery as a case study, the results showed about 10% energy efficiency increase in the site and a possible 33% energy efficiency increase if all recoverable waste heat sources were exploited. In a technical review on waste heat recovery from compression ignition engines, Chintala et al [3] showed that CI engines using organic Rankine cycle could operate within maximum thermal efficiency range of about 10–25%. Also, the review showed that for combined systems, thermal efficiency could range as high as 60–90%. In demonstrating the viability of ultra-low temperature waste heat recovery using organic Rankine cycle applied to dual loop data center applications, Ebrahimi et al [4] identified R134a and R245fa as the most appropriate working fluids. The use of superheaters to increase temperature at which waste heat is recovered was implemented successfully. Zhang et al [17] investigated a heat recovery chimney. Waste heat was captured from a steam power generation system. Their experimentation led to the conclusion that heat transfer rates at the condenser (water-air heat exchanger) depended on the size of the heat exchanger and air velocities at a given water flow rate and inlet temperature; and that the implementation of the heat recovery chimney will make the conventional steam power cycles more effective without additional environmental pollution. Bilge and Temir [18] investigated experimental and theoretical approach to heat recovery in air conditioning systems. The study used a plate heat exchanger in an air handling unit to recover heat from exhaust air for heating fresh air.

In recent studies involving the use of refrigerators for waste heat recovery, Srithar et al [19] investigated energy recovery from a vapour compression refrigeration system using humidification dehumidification desalination. The focus was on heat recovery for desalination. Babu et al [20] studied refrigeration using waste heat recovery from exhaust gas of an engine while Lu et al [21] investigated a novel hybrid refrigeration system for industrial waste heat recovery. Most heat recovery studies aimed to use recovered heat for refrigeration or industrial processes. Not much attention has been devoted to application of waste heat recovery for domestic use, particularly using waste heat recovered within a household to serve another household need. The present study addresses this gap as waste heat from a household refrigerator is utilized for another household activity – fabric drying. Hence, design and construction of a vapour compression cycle integrated R-134a refrigerator-waste heat recovery dryer for domestic drying of fabrics is presented. Drying of fabrics is done outdoors in most rural settlements in tropical countries, hence fabrics drying is always a challenge during rainy seasons. In this study, heat recovered from the condenser of a refrigerator was channeled into a drying chamber and its effectiveness for drying evaluated using fabrics. This study is important in Nigeria especially given the gross energy shortages and the need for indoor drying of fabrics particularly during rainy seasons. The application of vapour compression cycle to waste heat recovery for drying of fabrics is a novel work. While several works exist on application of vapour compression cycle towards many case studies, there is no evidence of application to drying of fabrics. This work is aimed at contributing to the body of knowledge by providing data on the effectiveness of a hybrid unit for both cooling using a refrigerator and drying of fabrics within a household.

## 2 Development of IRWHRD unit

**Figure 1:**
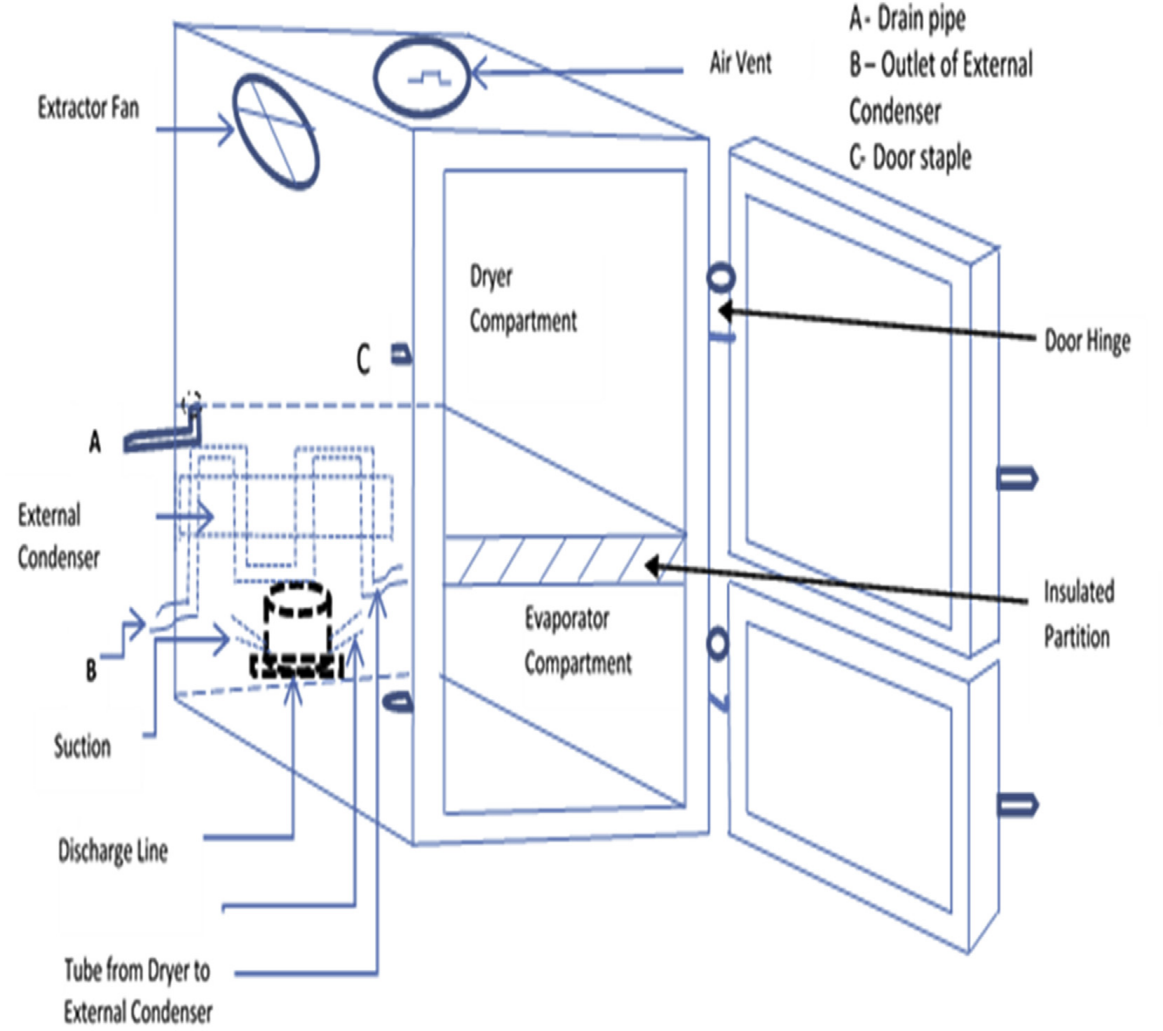
Schematic diagram of the configuration of IRWHRD.

**Figure 2:**
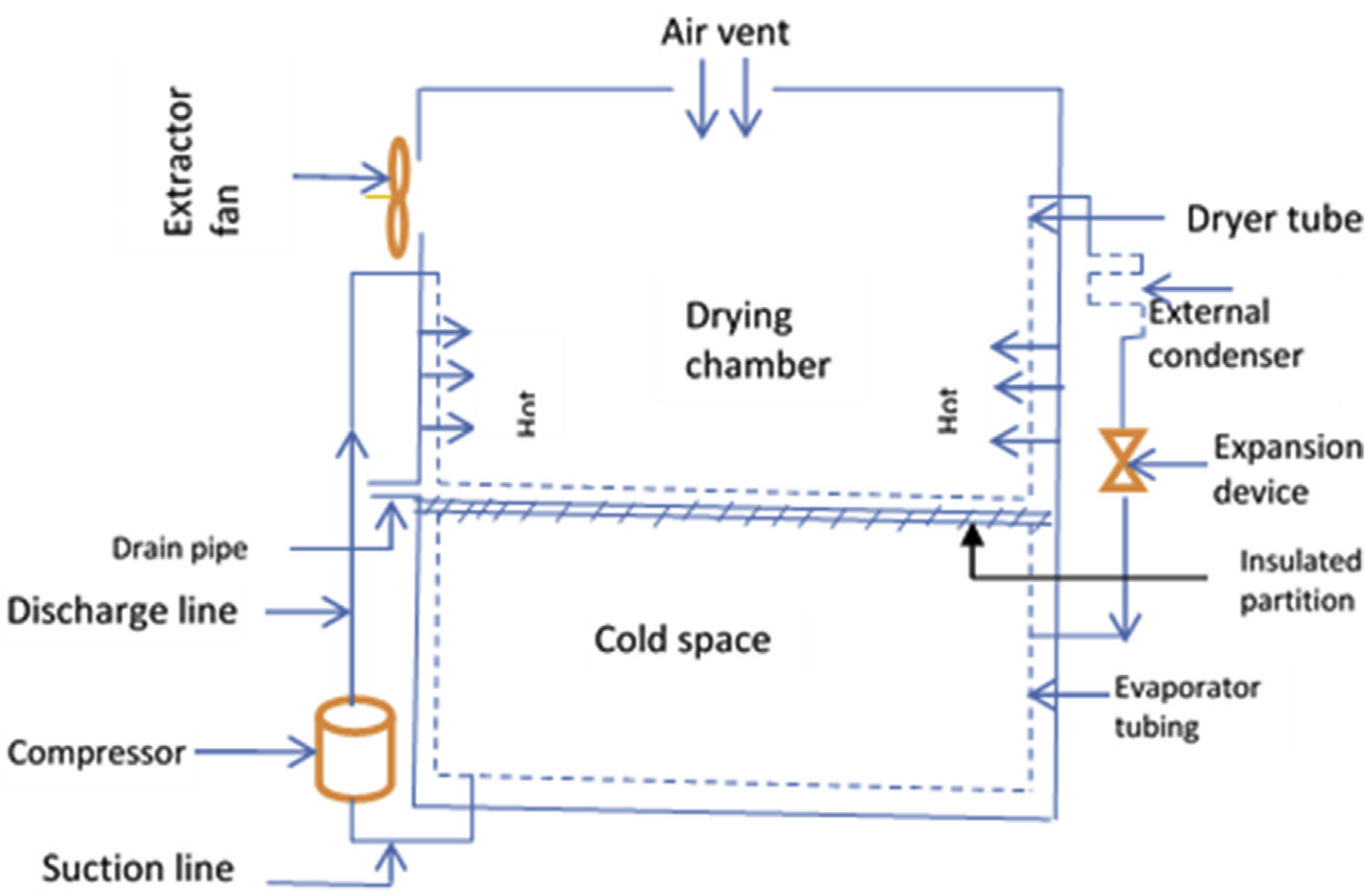
Major components of IRWHRD.

The configuration of the Integrated Refrigerator-Waste Heat Recovery Dryer (IRWHRD) is presented in Figure 1, while Figure 2 shows the component parts. In the IRWHRD, the dryer box acts as the internal condenser and experiences increases in the surrounding air temperature, while the sub cooling takes place in the external condenser. The throttle device is an expander made of capillary tube. The filter dryer is located at the beginning of the capillary tube and acts as a dehydrator to extract moisture from the refrigerant. The dryer is a rectangular box housing part of the condenser (of the refrigerator unit) and hence absorbs heat from the portion of the condenser it is housing. This leads to raised temperatures within the dryer, thereby attaining sufficient temperature for drying activity. The dryer box and its auxiliaries (extractor fan, air vent and drain pipe) form the dryer (drying chamber). The dryer has inner dimensions of width 0.5m, length 0.5m and height 100 cm. This is carefully chosen to enable the dryer handle conveniently the intended materials for drying. The evaporator is similarly 0.5m long and 0.5m wide.

### 2.1 Sizing of IRWHRD

**Figure 3:**
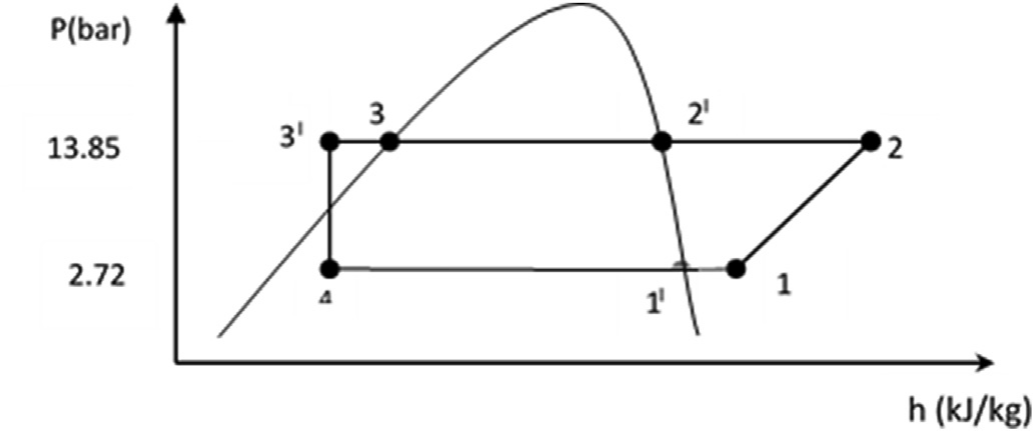
Schematic P-h diagram of IRWHRD.

Figure 3 shows a pressure-enthalpy (p-h) diagram [22] indicating all the heat transfer processes for a complete refrigeration cycle [12] as adopted for the IRWHRD. It consists of the following processes: compression (1-2), condensation (2-3^’^), expansion (3^’^-4) and evaporation (4-1). The following assumptions were made: (i) the compression process is considered isentropic, (ii) the condensation and evaporation processes occur at constant pressure and (iii) change in the enthalpy of the refrigerant liquid as it flows through the throttle device is negligible.

Dry superheated vapour enters the compressor and is compressed from state 1 to 2. At state 2 the refrigerant is superheated from T_2_^'^ to T_2_. During the compression process the work of compression and by implication the heat transferred to the refrigerant due to the compression process is estimated using the steady flow energy equation [23].

The following design equations were used in sizing the IRWHRD. The heat required for drying was estimated using Eq. (1):

(1) $$Q=\dot{m_{a}}c_{a}\Delta T$$

The mass flowrate of air is estimated using Eq. (2):

(2) $$\dot{m_{a}}=\rho_{a}{\text{A}}_{a\upsilon_{a}}$$

The product load is determined using Eq. (3):

(3) $$q_{wa}=m_{w}c_{pw}\Delta T_{w}+m_{wc}c_{pwc}\Delta T_{wc}$$

The system was designed such that the load should be removed in 16 h, hence the total quantity of heat due to the product and air change, Q_waa_ is given as:

(4) Qwaa=qwaa(16∗3600)

(5) $$\text{Where}\;q_{waa}=q_{wa}+q_{a}$$

Enthalpy of both inside air and outside air were estimated from ASHRAE psychrometric chart. Relative humidity expected in the refrigerated space was 90 % [22], environmental temperature in Owerri was averaged at 33 °C, while average environmental relative humidity for Owerri was 80 %.

In estimating the overall heat transfer coefficient of the walls (U_w_) of the system, Eq. (6) was used.

(6) 1Uw=1his+∑i=1n(xiki)+1hos

Heat gain into the cold space through the four vertical walls, Q_w1-4_, is given as:

(7) $$Q_{w1-4}=U_{w}\sum A_{w}\left(t_{o}-t_{i}\right)$$

Heat gain from the underside (bottom) of the system, Q_b_, is given as:

(8) $$Q_{b}=U_{b\;}*A_{b}*\left(t_{o}-t_{i}\right)$$

To estimate overall heat transfer coefficient of the partitions in the developed system, U_p_, Eq. (9) was used:

(9) 1Up=1hc+∑i=1n(xiki)+1he

Hence, heat gained through the partition of the dryer component, Q_p_, is given as:

(10) $$Q_{p}=U_{p}*A_{p}*\left(t_{c}-t_{e}\right)$$

In designing the tubing and fin area of the sub-cooled section, the sensible heat released by the refrigerant, Q_tf_, was estimated thus:

(11) $$Q_{tf}=U_{o}A_{tf}\left(T_{3}-{\dot{T}}_{3}\right)$$

Where the average overall heat transfer coefficient, U_o_, is given as:

(12) Uo=1(AsoAsix1hfc+Asoln(ro/ri)(2πkL33')+AsiAso(1/hso))

### 2.2 Refrigerator thermal design parameters

Tetrafluoroethane (R-134a) refrigerant was used in the study and the evaporator temperatures were 5 °C (for the cold space) and -2 °C (for the refrigerant). The saturation pressure corresponding to the evaporator (refrigerant) temperature was 2.72bar. Allowance for 5 °C superheat was provided for in the refrigerant along the suction line in order to prevent some refrigerant leaving the evaporator as liquid, which could lead to useful cooling potential being wasted. Superheating also forestalls liquid refrigerant arriving the compressor with the tendency to wash the lubricating oil from the walls of the cylinder, accelerating wear and damaging the valves or the cylinder head. Also, the dryer temperature was chosen as 50 °C [24], and the condenser (refrigerant) temperature was 52 °C, at least 19 °C above environmental temperature [25]. The saturation pressure corresponding to the condenser temperature is 13.85bar. For effective heat transfer between the condenser and the ambient (environmental) air, the condenser refrigerant was sub cooled at least 10 °C above the environmental (ambient) temperature [26]. Since the average environmental temperature within Owerri was 33 °C [27], the condenser refrigerant was sub cooled from 52 °C to 43 °C. The sub cooling was achieved using the ambient air. The heat required for drying is equivalent to the heat required to raise the ambient air temperature of 33 °C to the dryer temperature of 50 °C. Air was admitted into the drying chamber through the circular opening (air vent) of diameter 0.102 m located on the roof of the IRWHRD, while heat was added to the dryer to heat up ambient air from 33 °C to the desired dryer temperature at 50 °C. The refrigerant mass flowrate was determined using the principle that the heat released by the refrigerant in the condenser is equal to the heat required for drying by the dryer. Table 1 provides a summarized specification guide for the various components as designed.

**Table 1:** Design background specifications.

Compartment	Parameters	Values/Specification
A	Refrigerant	R-134a (Tetrafluoroethane)
	Refrigerant mass flowrate	0.001 kg/s
B	Dryer temperature	50 °C
	Length of dryer box	0.5 m
	Width of dryer box	0.5 m
	Height of dryer box	1.0 m
	Heat required in the dryer	0.1659 Kw
	Length of dryer hanging rod	0.5 m
	Gap between dryer roof and hanger rod	0.03 m
C	Cold space (evaporator box) temperature	5 °C
	Length of cold space	0.5 m
	Width of cold space	0.5 m
	Height of cold space	0.7 m
	Evaporator temperature	-2 °C
	Evaporator superheat temperature	5 °C
D	Condenser temperature	52 °C
	Condenser sub-cooled temperature	43 °C

### 2.3 Load estimation

Refrigeration or cooling load refers to the rate at which heat must be removed from the refrigerated space and material in order to produce and maintain the desired temperature conditions. The system was expected to cool about 40kg of water from the average environmental temperature of 33 °C to a desired product temperature of 5 °C. Basically, the evaporator is for cooling drinks but water was used as a reference material for the design. Sources of cooling load on the system included:

i. Heat flow into the refrigerated space from the surrounding air.
ii. Heat flow into the refrigerated space by warm outside air entering the space when the door was opened (infiltration). This is governed by the number of air changes per unit time
iii. Heat taken out from the product (which in this case was 40kg of drinking water) in order to cool it from its initial temperature to the desired storage temperature. This is called product load.

The equipment was designed to run for 16 h within which period it would have cooled the product from the initial ambient condition (33 °C) to the desired condition (i.e. 5 °C).

Air change simply means the replacement of the more dense cold air in the refrigerated space by warm outside air. The heat which must be removed from this warm outside air to reduce its temperature and moisture content to the space design conditions becomes a part of the total cooling load on the equipment. Air changes occurring in the refrigerated space are caused mainly by infiltration through door openings.

The following assumptions were used in evaluating the air change load:

- Relative humidity expected in the refrigerated space was 90% [22].
- Environmental temperature = 33 °C
- Environmental relative humidity for Owerri = 80% average
- From average air change per 24 h chart, air change per 24 h for storage compartments above 0 °C due to door openings and infiltration (on average usage) was taken as: 60 air changes per 24 h [22].

From ASHRAE psychrometric chart: enthalpy of inside air (at 90% RH and 5 °C) = 18.01 kJ/kg; enthalpy of outside air (at 80% RH and 33 °C), = 111 kJ/kg; air density at 33 °C environmental temperature (average) = 1.2 kg/m^3^ [28].

Wall gain load is a measure of the heat flow rate by conduction through the walls of the refrigerated space from the outside air to the inside. There is heat gain into the evaporator through the four vertical walls, underside or bottom wall and the dryer compartment. The following design material selection was utilized: Outer wall was made of 0.8mm thick galvanized steel plate; Inner wall was made of 0.6 mm thick aluminum plate; Insulation material = 5cm thick polyurethane board. The polyurethane board was sandwiched between the outer galvanized steel plate and the inner aluminum plate. In order to effectively determine the wall heat load, the materials specification of the refrigerator used are shown in Table 2.

**Table 2:** Material specifications; the k values are read from [28].

Material	K value (W/m^2^k)
Galvanized steel plate	42.98
Aluminum plate	203.94
Polyurethane foam	0.03

The overall heat transfer coefficient for the bottom, U_b_ is the same with that of the vertical walls. This is because the bottom of the IRWHRD was made of the same materials as the walls. The dryer box and the evaporator box are separated by an insulated partition (separator). The partition was of the same dimensions as the bottom of the evaporator box. Therefore the area of the partition, A_p_ = 0.5 × 0.5 = 0.25m^2^. The partition was well insulated in order to reduce the heat flow from the dryer box to the minimum. The partition was made up of 5cm of polyurethane board sandwiched between 0.6mm aluminum plates (with each plate on the either side of the polyurethane board). Other parameters for the partition included average temperature in the dryer box side of the partition = 50 °C; average temperature in the evaporator box side of the partition = 5 °C. The temperature gradient between the dryer and the evaporator boxes caused heat flow from the dryer to the evaporator.

Total refrigeration (cooling) load, Q_R_, is the summation of heat gain through the partition, Q_p_, heat gain through the underside (bottom), Q_b_, heat gain through the four vertical walls, Q_w1-4_, and heat gain from product and air change loads, Q_waa_

(13) $$\text{That}\;\text{is},\;Q_{R}=\sum \left(Q_{waa}*Q_{w1-4}*Q_{b}*Q_{p}\right)$$

It is common practice to add between 5% and 10% of the cooling load as factor of safety. For this work, a 5% factor of safety was added to the total cooling load and approximated to the next whole number. A summary of the load estimation is presented in Table 3.

**Table 3:** Summary of load estimation.

Parameter	Value
Product load, q_wa_	4751.7 kJ
Air change load, q_a_	1956.7 kJ
Total quantity of heat due to the product and air change, q_waa_	6708.4 kJ per 24 h
Removed load in 16 h, Q_waa_	116 W
Heat gain through the four vertical walls, Q_w1-4_	21.56 W
Heat gain from the underside (bottom), Q_b_	3.85
Heat gain through the partition, Q_p_	5.96 W
Total refrigeration load, Q_R_	150 W

### 2.4 Compressor capacity

According to Desai [29], the selection of compressor for a given application depends on the required refrigeration capacity, the design saturated suction temperature and the design saturated discharge temperature. Hence the selection of compressor for this work was based on the refrigeration capacity. The refrigeration capacity for this work is 150W as determined earlier. Hence a compressor that is rated at 150W power was selected.

### 2.5 Design specifications of the evaporator

Copper pipe was selected for this work as it is compatible with all halogenated refrigerants including R-134a. It is also resistant to corrosion, cheap and easy to install [25]. In designing for the evaporator tubing, the following choice and assumptions were made;

i. Type L (medium thick wall) copper tube of 8mm (0.008m) outer diameter and 6.3mm (0.0063m) internal diameter was selected based on American Standard Safety Code for Mechanical Refrigeration Code (CSA B52)
ii. The refrigerant was to enter the evaporator tubing at 7 °C below the saturation temperature of the 5 °C desired in the cold space [25]. This implied that the refrigerant entered the evaporator tube at -2 °C which corresponds to 2.72bar.
iii. The refrigerant was to be superheated by 5 °C towards the end of the evaporator tube. The 5 °C superheat was allowed in the refrigerant along the suction line in order to prevent some refrigerant leaving the evaporator as liquid, which would lead to useful cooling potential wasted. Superheating also forestalls liquid refrigerant arriving the compressor with the tendency to wash the lubricating oil from the walls of the cylinder, accelerating wear and damaging the valves or the cylinder head.

The pipe (tubing) was wound inside the evaporator compartment and covered with aluminum plate. The tubing was assumed to make a good thermal contact with the aluminum plate, hence the aluminum plate provided a larger surface for heat transfer by acting as a fin. Summary Design Specifications of the Evaporator is as in Table 4.

**Table 4:** Design specifications of the evaporator.

Compartment	Parameters	Value/Specification
A	Tube material	Copper
	Inner diameter	6.3 mm
	Outer diameter	8.0 mm
	Length of tube	12.67 m
B	Length of evaporator box	0.5 m
	Width of evaporator box	0.5 m
	Height of evaporator box	0.7 m
	Insulation material	Polyurethane foam
C	Evaporator inner wall material	Aluminium
	Thickness of inner wall material	0.6 mm
	Outer wall material	Galvanized steel plate
	Thickness of outer wall material	0.8 mm

### 2.6 Design specifications of the condenser

The condenser was desired to release 50 °C heating temperature into the dryer box. In designing for the condenser tubing, the following choice and assumptions were made;

i. Type L (medium thick wall) copper tube of 4.5mm (0.0045m) outer diameter and 2.8mm (0.0028m) internal diameter was selected based on American Standard Safety Code for Mechanical Refrigeration Code (CSA B52)
ii. The refrigerant temperature in the condenser tubing was 52 °C based on the recommendation of Althouse et al [25] which suggested that the temperature of the refrigerant in an air cooled condenser is approximately 17 °C–19 °C warmer than the environmental temperature. Hence choosing 19 °C above average environmental temperature in Owerri of 33 °C [27], the condenser temperature for this work therefore was 52 °C.
iii. The refrigerant was to be superheated by 5 °C at the exit section of the evaporator tubing in order to prevent some refrigerant leaving the evaporator as liquid, which is useful cooling potential wasted [29]. Superheating also forestalls liquid refrigerant arriving the compressor with the tendency to wash the lubricating oil from the walls of the cylinder, accelerating wear and causing damage to the valves or the cylinder head. With the 5 °C superheat, the entry temperature at the condenser therefore, is the sum of the superheat and the condenser temperature valued at 57 °C.
iv. For effective heat transfer between the condenser and the ambient air, the condenser refrigerant was sub-cooled at least 10 °C above the ambient temperature [26]. Since the environmental temperature within Owerri is 33 °C [27], the condenser refrigerant therefore was sub-cooled to 43 °C (environmental temperature plus 10 °C).
v. The condenser tubing wound round the drying chamber (dryer) was assumed to make a good thermal contact with the aluminum plate covering it. Hence the aluminum plate provided a larger surface for heat transfer, and therefore acted as a fin.

A summary of the design specifications for the condenser is presented in Table 5.

**Table 5:** Design specifications of the condenser.

Compartment	Parameters	Value/Specification
A	Tube material	Copper
	Inner diameter	2.8 mm
	Outer diameter	4.5 mm
	Length of tube for superheat and condensation	9.38 m
B	Length of condenser box (drying chamber)	0.5 m
	Width of condenser box (drying chamber)	0.5 m
	Height of condenser box (drying chamber)	1.0 m
	Insulation material	Polyurethane foam
C	Condenser (drying chamber) inner wall material	Aluminium
	Thickness of inner wall material	0.6 mm
	Outer wall material	Galvanized steel plate
	Thickness of outer wall material	0.8 mm
D	Length of tube for sub-cooling	1.0 m

### 2.7 Design of the tubing and fin area of the sub-cooled section

The tubing in the sub-cooled region was wound onto a galvanized steel plate in serpentine form and mounted on the rear of the IRWHRD. The plate provided a larger surface area for heat removal from the tubing to the surrounding environment, thus playing the function of a fin. Two convection heat transfer coefficients were prevalent in the sub-cooled region - the convection heat transfer coefficient prevalent inside the refrigerant, h_fc_, evaluated as 0.50kW/m^2^K; and the convection heat transfer coefficient acting on the bare tube, h_so_. This is called the outside wall convection heat transfer coefficient; evaluated as 22.71W/m^2^K [22].

The refrigerant was completely liquid in the sub-cooled region. A total surface area, A_scT_ (of tubing and fin) was required to cool the refrigerant further from T_3_^'^ (43 °C) to environmental temperature, T_o_ (33 °C).

The plate upon which the sub-cooled tubing was wound is galvanized steel plate with a rectangular shape. The choice of the galvanized steel plate was influenced by the following reasons:

• According to the American Galvanizers Association, galvanized steel resists corrosion up to 100 times better than uncoated steel. The resistance of the galvanized steel to corrosion was a major criterion for its choice as the outer wall material of the IRWHRD.
• The galvanized steel also gives good surface appearance and allows for a higher quality finish when painted.
• The zinc coating on the galvanized steel is resistant to cracking and loss of adhesion when the steel is formed into any shape.
• Galvanized steel is extremely durable and resistant to scratches from abrasion.
• The tensile strength of galvanized steel also favours its choice as the outer wall material for the IRWHRD.

Additionally, galvanized steel plate was chosen for fabricating the plate fin due to its heat conduction qualities. Galvanized steel plate can extract the heat in the sub-cooled tubing attached to it and convect it away to the surrounding air. The thickness of the galvanized steel, t_f_ used for the plate fin as chosen is 0.5mm. The 0.5mm plate, a lighter gage, was chosen so as to avoid excessive weight.

A summary design specification of the sub-cooled tubing and fin are as in Table 6.

**Table 6:** Design specifications of the sub-cooled tubing and fin.

Compartment	Parameters	Value/Specification
A	Tube material	Copper
	Inner diameter of sub-cooled tubing	2.8 mm
	Outer diameter of sub-cooled tubing	4.5 mm
	Number of tube arms	4
	Length of each tube arm	0.14 m
	Number of tube elbows	3
	Diameter of each bent tube elbow	4 cm
B	Plate fin material	Galvanized steel
	Length of plate fin	18 cm
	Height (breadth) of plate fin	11 cm
	Thickness of plate fin	0.5 mm

### 2.8 Fabrication procedure

**Figure 4:**
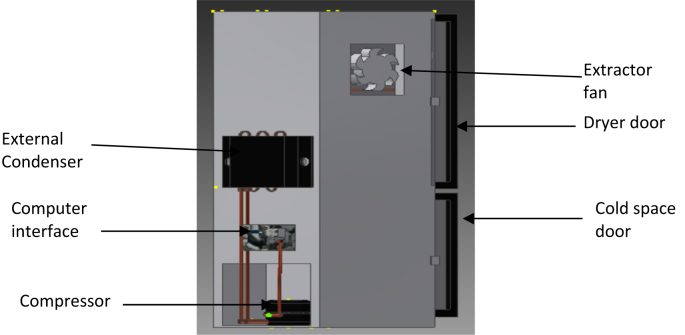
Assembly of the IRWHRD

The instruments used for the fabrication of the IRWHRD include: tool box (containing cutters, pliers, wrenches etc.), oxy-acetylene welding set, pipe bending instrument, guillotine machine (for folding sheet metals) and riveting machine. Other equipment and gages include: thermometers, gage manifold set, evacuating pump, leak detector, anemometer, sling psychrometer, clamp-on ammeter, digital spring balance and extractor fan. Consumables include: brass rods, easy flow rods, soldering flux, oxy-acetylene gas and sheet metals (aluminum and galvanized). The assembly of the IRWHRD is shown in Figure 4.

### 2.9 Refrigerant charging

Vacuuming and leak testing were undertaken before refrigerant charging into the system was carried out. Vacuuming was done in order to remove air and moisture from the system, while leak testing was done to check for leakages in the system. The system was evacuated via a manifold gage and vacuum pump arrangement. The vacuum was drawn down to 100 microns. The compound gage (of the manifold gage) hand valves was then locked while noting the reading on the gage. A zero upward movement of the gage pressure indicated that the system was free of moisture. Similarly, a zero downward movement of the gage pressure indicated that there was no leakage in the system. After the evacuation, the compound gage hose was connected to the compressor service suction valve. The compound gage valve was then closed. The centre hose of the manifold gage was attached to the refrigerant cylinder and air was then purged from the hose. The valve of the compound gage hose was opened to allow refrigerant flow into the compressor and charged to required pressure. Gas charging was used and this was mainly to enable gas volume to be accurately controlled in accordance with the readings of suction and discharge gages. A clamp-on ammeter was mounted on the compressor to indicate the power drawn by the compressor.

### 2.10 Compressor installation and leakage problems

The installation of the compressor and the initial leakage test via manifold gage showed no leakage in the system. However, as the compressor started pumping refrigerant through the system, there was persistent smear of oil at the joint between the filter dryer and the external condenser tube. This was physically observed. However, the application of soap solution at the joint showed that there was minute bubble which was indicative of leakage. The system was evacuated again and recharged. The oil smear still showed up at the same joint after the recharge. The minute leakage at the external condenser-filter dryer joint was later traced to wrong positioning of the filter dryer which was found to be tilted a little above the horizontal. This positioning of the filter dryer coupled with the resistance to flow by the capillary tube attached to it, impeded free flow of the liquid refrigerant coming from the external condenser. The degree of hotness felt at the filter dryer and capillary tube also showed that there was excessive pressure build up at the external condenser as a result of the restriction to refrigerant flow. This problem of persistent oil smear with the accompanying minute leakage was solved by tilting the filter dryer slightly below the horizontal so as to enable the refrigerant flow under gravity as it flowed from the condenser through the capillary tube. More so, to forestall the hotness of the refrigerant flowing through the capillary tube (to the evaporator), the capillary tube was wound on a section of the suction line. This provided a heat exchange between the capillary tube and the suction line thereby sub-cooling the refrigerant and minimizing the formation of flash gas in the capillary tube before the refrigerant flowed into the evaporator. The vapour in the suction line also benefited from the heat exchange by getting superheated which in turn increased the refrigerating effect of the refrigerator.

### 2.11 Instrumentation

**Figure 5:**
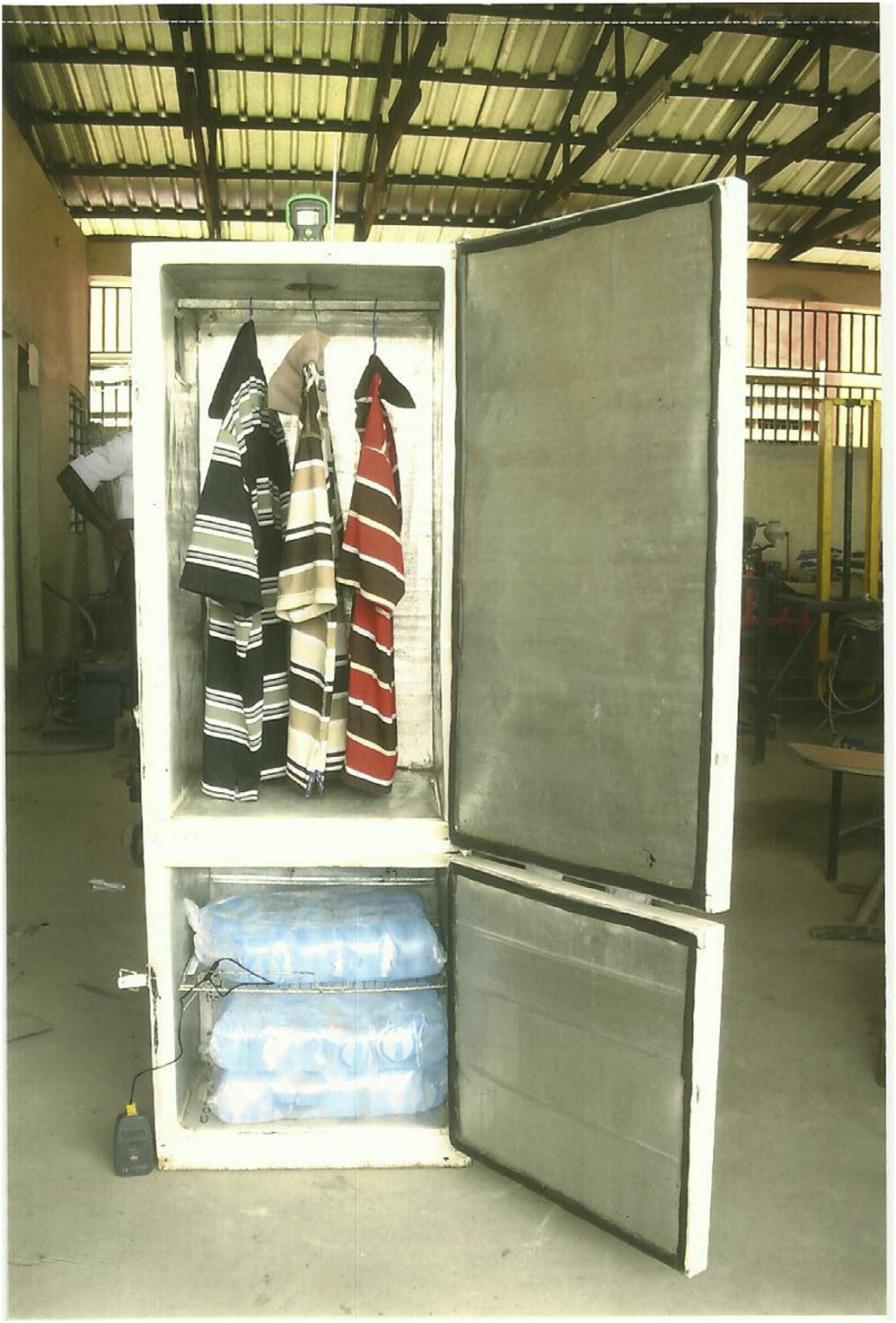
Developed IRWHRD with samples of drying cloths.

The major challenge of test running the IRWHRD equipment was that of ensuring uninterrupted operation for at least six hours within which period the system would have attained observable stable temperature at the cold space (evaporator box) and drying chamber (dryer). This was hard to achieve due to the epileptic nature of power supply in Nigeria. An alternative source of power was provided by the use of 2 kVA generating set which could operate for at least ten hours at full tank fuel supply. In this way, the IRWHRD temperature variations and drying rates were monitored uninterrupted. Readings were taken at regular intervals of 15 min for drying rate experiments and 30 min for temperature monitoring. The temperature of the evaporator box (cold chamber), was monitored by the use of k-type Mastech MS 6500 digital thermometer with error limit of ±0.5%. The thermometer has two major parts, namely, the sensitive stainless probe and the digital box. The probe was connected to the digital box via a certain length of cable. During experimentation, the probe was put inside the evaporator while the digital box was located outside the evaporator box. On closing the door of the evaporator box, the soft gaskets on the door edges held the cable connecting both the digital box and the probe in place. The soft door gaskets maintained an air tight hold on the connecting cable and thus prevented or greatly reduced air ingress into the evaporator. The cable was closed in between the door gaskets in such a way that the probe was hanging on the air inside the evaporator box without touching its floor or side walls. Keeping the probe in this position ensured that the thermometer only indicated the uniform air temperature prevalent in the evaporator box. The temperature of the evaporator box was read on the Mastech digital box every thirty minutes. In this way, the Mastech digital thermometer ensured continuous observation of the evaporator temperature without opening its door; as opening its door would admit outside air into it, which would interfere with its cooling rate. Installed at the back of the IRWHRD was the transmitter-receiver box. This was interfaced with the computer to remotely display temperature variations in the evaporator. Weight loss of the product inside the drying chamber was also constantly monitored by the use of digital hanging scale. The digital hanging scale had three major parts - the stainless hook at one end, the hanger (handle) at the other end and the digital box (in between the two ends) which displayed the mass of cloths hung on the hook in kilograms. For continuous operation, the hook of the hanger was located inside the drying chamber through the air vent on the roof of the drying chamber while both the digital box and handle were located outside the drying chamber. The stainless hook carried the wet cloth to be dried, while the handle served to support the whole weight of the hanging scale and wet cloth on any platform. The platform upon which the handle of the digital scale was hung was a short 20cm long 8mm diameter steel rod which was supported on two stands improvised from wooden blocks and which were standing on top of the roof of the drying chamber. The reading of the weight (kg) displayed on the digital scale was taken every 15 min without opening the door of the drying chamber. Hence, periodic amount of moisture removal from the cloth was observed without opening the door of the dryer. This was to avoid influx of outside air which may reduce the temperature of the dryer. The drying temperature was observed by installing 100 °C mercury in glass thermometer. The bulb of the thermometer was held in a slit cut in a thick carton paper sheet. The paper sheet, 15 cm × 15 cm was placed loosely on the 10cm diameter air vent located on the roof top of the IRWHRD in such a way as not to hinder free flow of outside air into the dryer. Both the hook of the hanging digital scale and the bulb of the mercury in tube thermometer were passed through the carton paper sheet placed on the 10cm diameter air vent into the drying chamber. In this position, the bulb of the thermometer was in contact with the hot air being circulated in the drying chamber hence continuously reading the drying chamber temperature. At the back of the IRWHRD, a transmitter-receiver box was installed. This was interfaced with the computer to display temperature variations in the drying chamber. The computer monitored the temperatures of the drying and cold chambers from within 30m radius to the IRWHRD. Figure 5 shows the photograph of the developed IRWHRD with samples of drying cloths.

## 3 Results and discussion

### 3.1 Presentation of results

**Figure 6:**
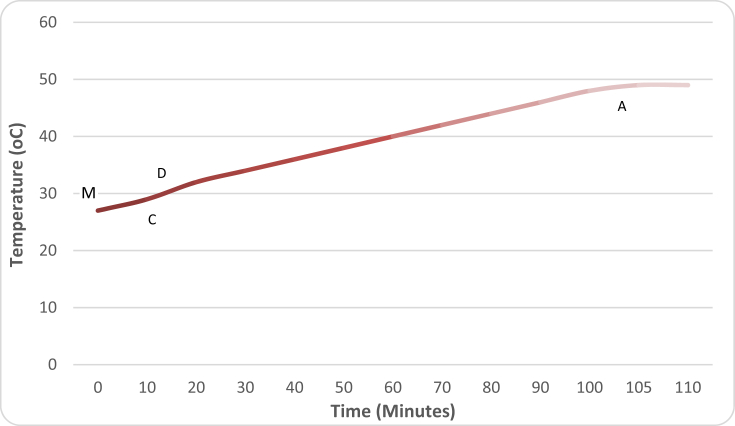
Dryer temperature variations under unloaded condition (extractor fan off).

**Figure 7:**
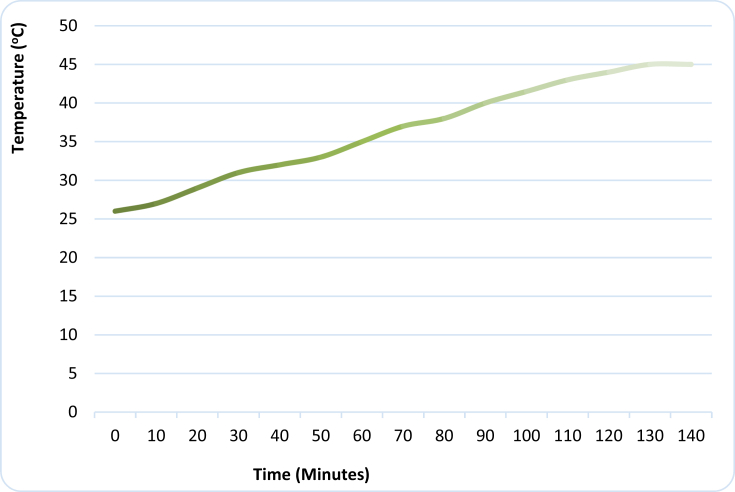
Dryer temperature variations under unloaded condition (extractor fan switched on).

**Figure 8:**
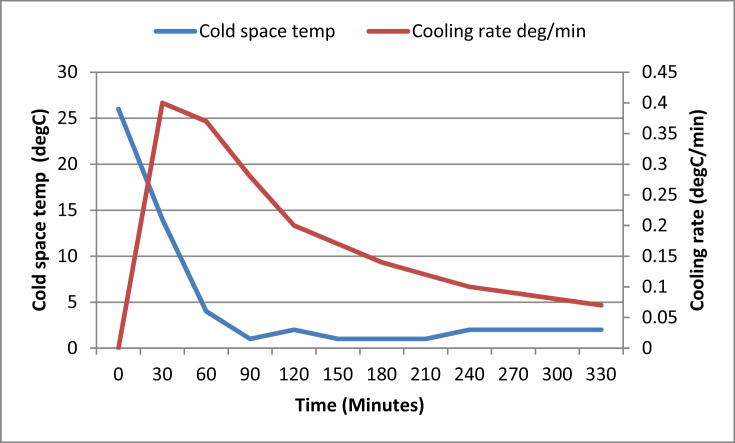
Variation of cold space temperature and cooling rate with time (cold space unloaded).

**Figure 9:**
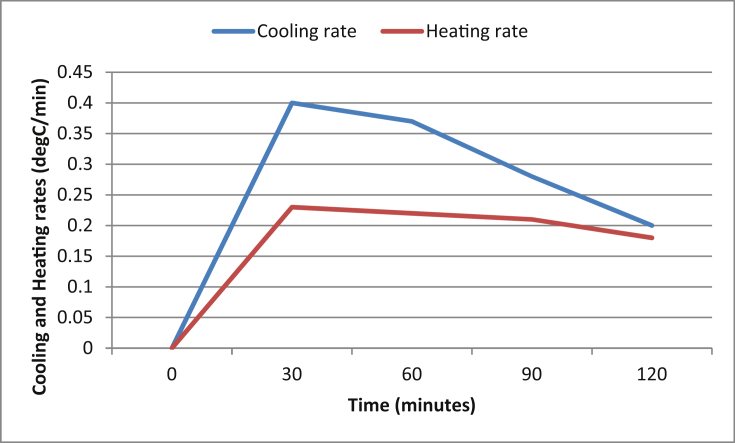
Cooling and heating rates when the cold space was not loaded.

**Figure 10:**
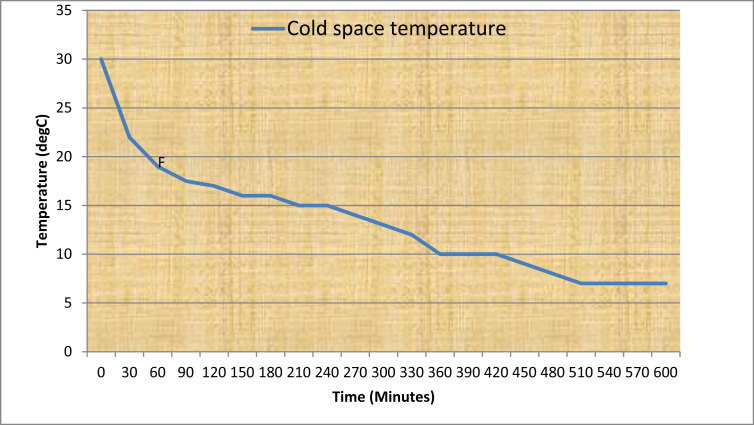
Cold space temperature and cooling rate for ten hours (cold space loaded; extractor fan on).

Table 7 shows the operating parameters of the developed IRWHRD. Results of experimentation of the IRWHRD when the cold space was not loaded with design product (water) are presented in Figures 6 and 7; Figure 8 shows the variation of cold space temperature and cooling rate with time, while Figure 9 illustrates the results for the cooling/heating operations under unloaded conditions. When the cold space was loaded with 42kg of the design product (water) and the experiments repeated, the result is presented in Figure 10.

**Table 7:** System operating parameters.

T_eva_ ^o^C	T_dryer_^o^C Max	Drying Temp ^o^C	ṁ_R_ kg/s	Q_dryer_ (Kw)	Drying air velocity	Q_eva_ (Kw)
2 and 7	49	45 and 47	0.001	0.1659	0.39	0.15

### 3.2 Drying temperature without load

The drying chamber temperature without load was observed at intervals of 10 min up to a total drying period of 110 min using type-k digital thermometer. After 110 min, the extractor fan was switched off. This was to ensure that the temperature profile of the dryer was monitored without air flow interference from the extractor fan. The drying chamber temperature increased from an initial value of 27 °C to a maximum value of about 50 °C (Figure 6). Near-steady drying chamber temperature increases commenced around 10 min after commencement of experimentation; which means that the initial period was used in heating the chamber to temperatures above the ambient. The asymptotic profile at about 50 °C horizontal indicates that the drying chamber temperature attained its maximum value after about 105 min of the system operation. The heat dissipated in the drying chamber is the heat from the superheat and condensation regions of the condenser. Figure 6 is a reflection of the temperature and pressure changes of the refrigerant in the condensing unit. Before the commencement of compression, the refrigerant in the compressor can be assumed to be at the same temperature with that of the surrounding environment. As the compressor starts, it compresses the refrigerant vapour almost at a constant temperature (MC on Figure 6) for the first ten (10) minutes. This corresponds to the warm up stage. At this stage temperature differential between the refrigerant and the condensing medium (air in the drying chamber) is not much and hence, there is insignificant heat transfer between the two media. The vapour pumped into the condenser will not condense at this stage because its pressure is below its saturation vapour pressure. As the compressor pumps more vapour into the condenser after about twenty (20) minutes of operation, there was a noticed sharp temperature rise (CD on Figure 6). Further pumping of vapour into the condenser led to continuous increases in temperature of the dryer from point (D) to point (A) at 49 °C. After point (A) the vapour temperature remains constant. Point (A) is the temperature at which the required rate of heat transfer between the refrigerant vapour and the condensing medium (air in the drying chamber) occurs. The maximum temperature (49 °C) of the dryer was recorded with the extractor fan switched off. Figure 7 shows the temperature profile of the drying chamber in unloaded condition with the extractor fan switched on. The maximum temperature observed in the dryer with the extractor fan in operation was 45 °C. Further experimental runs showed that it took about 130 min for the dryer to get to a temperature of 45 °C (Figure 7). The reduction in the maximum dryer temperature from 49 °C to 45 °C is as a result of increased heat transfer (between the drying chamber and the outside environment) induced by air flow of the fan. Recall that the drying chamber (dryer) design temperature is 50 °C. Given that the maximum temperature observed in the dryer was 49 °C while the dryer design temperature was 50 °C, there was a 1 °C deviation in the dryer design. Therefore, dryer temperature deviation was 2% using Eq. (14):

(14) Dryertempdeviation=[(Designdryertemp−Actualdryertemp)Designdryertemp]

### 3.3 Analysis of cooling operations

#### 3.3.1 IRWHRD operations (unloaded cold space)

(a) Evaporator Temperature: Temperature readings were taken every thirty minutes on the type-k digital thermometer. Cold space temperature decreased steadily to 1 °C within the first 90 min (Figure 8). It later stabilized at 2 °C after 240 min of operation. Since the cooling chamber design temperature is 5 °C, and the actual lowest stable temperature in the evaporator as read from the type-k digital thermometer is 2 °C, it then means that the difference between the design and actual cold space temperature is 3 °C.
(b) Cooling Rate, C_r_ of the Cold Space under Unloaded Condition: Cooling rate curve was derived from the cold space temperature by dividing the cold space temperature change by the time taken for the corresponding temperature change. The cooling rate defined in this case as the change of temperature of the cold space under unloaded condition over time, was evaluated using Cooling rate, C_r_:

(15) Cr=(T0−Ti)(ti−t0)

Where T_o_ is the initial temperature of the cold space before cooling operation. It is the same as the prevailing environmental temperature. T_i_ is the temperature of the cold space at any given time, while t_i_ and t_o_ are the times corresponding to T_i_ and T_o_ respectively. The time before the commencement of the cooling operation, t_o_, is taken as zero. Hence from Eq. (15), when T_i_ = T_o_, C_r_ = 0.

Figure 8 shows the curve of the cooling rate under unloaded condition. It shows that the cooling rate was highest within the first 30 min, and tended to zero after about 24 h if the cold space temperature remained at 2 °C.

(c) Heating and Cooling Rates under Cold Space Unloaded Condition

The rate of heating and cooling (under unloaded condition) in the drying chamber and cold space are shown in Figure 9. The rate at which the cold space was cooling down is faster than the rate at which the drying chamber was heating up. Both heating and cooling rates were highest at the 30th minute of operation. However, there is almost an equal rate for both the cooling and heating after about 120 min of operation when the dryer peaked at 49 °C and the cold space stabilized at 1 °C. This connotes a state of equilibrium where the rate at which heat was absorbed in the cold space is equal to the rate at which heat was rejected in the dryer.

#### 3.3.2 IRWHRD operations (loaded cold space)

The cold space of IRWHRD was designed to cool 40kg of water down to 5 °C within 16 h of operation. The equipment was test run with the cold space loaded with 42kg of sachet water. The dryer was loaded with a fabric material. The experimental run lasted for six hours from 12.43pm to 18.43pm with the fabric material drying within the first two and half hours and the cold space temperature cooling down to 12 °C. The ambient relative humidity of the period as measured with type k environmental meter with error limit of ±0.5% ranged between 51% and 54%, giving an average RH of 52.5%, while the ambient temperature decreased from 34 °C to 32 °C. The cold space temperature and cooling rate profile is shown in Figure 10.

#### 3.3.3 Comparison with other test results

The SMER obtained in this work is lower than those from existing literature (Table 8). Hodgett [30] reported that the SMER for heat pump dryer is in the range of 1.0–4.0 kg/kW.hr, whereas the SMER for a conventional dryer is in the range of 0.2–0.6 kg/kW.hr. Baines [31] concluded that using heat pumps in dryers reduces the energy loss; he also obtained SMER in the range of 1–6 kg/kW.hr. The low SMER of 0.19–0.22 kg/kW.hr recorded in this work was attributable to the extra energy consumed by the extractor fan and the fact that the air used for the drying was not dehumidified before introducing it into the drying chamber, especially as the test periods for this work were periods when relative humidity ranged between 79 and 87%. This means that the air used for the drying process was itself laden with moisture which reduced its ability to extract good amount of moisture from the drying material. This phenomenon is typical of the rainy season in Nigeria. Adapa and Schoenau [32] recorded average SMER values for specialty crops in the range of 0.06–0.61kg/kWhr for agricultural products. This is lower than the SMER for this work as a result of the fact that agricultural products have lower moisture removal rate due to the fact that they have smaller surface area to mass ratio, meaning that their surface area exposed to drying air is much smaller than that of clothing material.

**Table 8:** Comparisons with other experimental results.

Waste Heat Recovery System	SMER (kg/kW.hr)
IRWHRD	0.19–0.22
Heat Pump Dryer [30]	1–4
Heat Pump Dryer [31]	1–6
Heat Pump Dryer for specialty crops [32]	0.06–0.61

## 4 Conclusion

A novel unit of Integrated Refrigerator- Waste Heat Recovery Dryer was successfully designed, fabricated and tested. The IRWHRD consists of two chambers, namely the heating (drying) and cooling chambers. Maximum temperatures of 49 °C and 2 °C were achieved in the heating and cooling chambers respectively during pre-loading test of the cold space. The amount of energy delivered by the condenser for drying operations was approximately 0.17 kW. Wetted cotton fabrics (clothes) were dried in the drying chamber at 45 °C–47 °C. The SMER values obtained were in the range of 0.19–0.22 kg/kWhr. Theoretical (maximum) COP of 10.84 was obtained when only the work input to compressor was accounted for through thermodynamic analysis. Lower COP of 10.09 was recorded when the work input of both the extractor fan and compressor was accounted for through direct measurement of the energy consumed. This was referred to as COP_ws_ (COP of the whole system).

## Declarations

### Author contribution statement

Ebenezer I. Onyeocha: Conceived and designed the experiments; Performed the experiments; Analyzed and interpreted the data; Contributed reagents, materials, analysis tools or data; Wrote the paper.

Kevin N. Nwaigwe, Nnamdi V. Ogueke & Emmanuel E. Anyanwu: Conceived and designed the experiments; Analyzed and interpreted the data; Contributed reagents, materials, analysis tools or data; Wrote the paper.

### Funding statement

This work was supported by the Federal University of Technology Owerri Ph.D. research fellowship and 10.13039/501100008895TETFUND staff development support.

### Competing interest statement

The authors declare no conflict of interest.

### Additional information

No additional information is available for this paper.
